# Smoking and pancreatic cancer: a sex-specific analysis in the Multiethnic Cohort study

**DOI:** 10.1007/s10552-022-01637-z

**Published:** 2022-10-17

**Authors:** Inger T. Gram, Song-Yi Park, Lynne R. Wilkens, Loïc Le Marchand, Veronica Wendy Setiawan

**Affiliations:** 1 Department of Community Medicine, Faculty of Health Sciences, UiT The Arctic University of Norway, Postboks 6050 Langnes, 9037 Tromsø, Norway; 2 Population Sciences in the Pacific Program, University of Hawai’i Cancer Center, Honolulu, HI USA; 3 Department of Population and Public Health Sciences, Keck School of Medicine and Norris Comprehensive Cancer Center, University of Southern California, Los Angeles, CA USA

**Keywords:** Cohort studies, Multiethnic Cohort (MEC) Study, Pancreatic cancer, Smoking, Smoking cessation

## Abstract

**Purpose:**

To examine whether the detrimental smoking-related association with pancreatic cancer (PC) is the same for women as for men.

**Methods:**

We analyzed data from 192,035 participants aged 45–75 years, enrolled in the Multiethnic Cohort study (MEC) in 1993–1996. We identified PC cases via linkage to the Hawaii and California Surveillance, Epidemiology, and End Results Program cancer registries through December 2017.

**Results:**

During a mean follow-up of 19.2 years, we identified 1,936 incident PC cases. Women smokers smoked on average less than men smokers. In multivariate Cox regression models, as compared with sex-specific never smokers, current smokers had a similar elevated risk of PC for women, hazard ratio (HR) 1.49 (95% CI 1.24, 1.79) and as for men, HR 1.48 (95% CI 1.22, 1.79) (*p*_heterogeneity_: 0.79). Former smokers showed a decrease in risk of PC for men within 5 years, HR 0.74 (95% CI 0.57, 0.97) and for women within 10 years after quitting, HR 0.70 (95% CI 0.50, 0.96), compared with their sex-specific current smokers. Both sexes showed a consistent, strong, positive dose–response association with PC for the four measures (age at initiation, duration, number of cigarettes per day, number of pack-years) of smoking exposure among current smokers and an inverse association for years of quitting and age at smoking cessation among former smokers (all *p*_trend_’s < 0.001).

**Conclusion:**

Although MEC women smoke on average less than their men counterparts, the smoking-related increase in PC risk and the benefits of cessation seem to be of similar magnitudes for women as for men.

**Supplementary Information:**

The online version contains supplementary material available at 10.1007/s10552-022-01637-z.

## Introduction

Worldwide pancreatic cancer (PC) is the 12th most common cancer, but due to its poor prognosis PC is the seventh leading cause of cancer deaths for both sexes [1]. Europe, North America, and Australia/New Zealand have the highest incidence rates of PC in the world [2]. In the USA, the incidence of PC continues to increase for both men and women. The PC incidence ranks as number ten among men and eight among women, while PC ranks as number four for cancer deaths for both sexes [3]. Recently, the PC incidence in the USA was predicted to increase by as much as 50%, from 2018 to 2040 [4].

Although the causes of PC are still insufficiently understood, many risk factors have been identified, as well as suggested in recent reports. Smoking is often considered the most harmful risk factor for PC. Due to the prevalence of smoking and the strength of the association with PC, smoking is likely to explain some of the international variations and sex differences [1, 4–9].

In the 1980’s, smoking was established as a cause of PC [10], while smoking cessation was recognized as reducing the risk of PC in 1990 [11]. Later expert reports from the International Agency for Research on Cancer [12, 13] and the US Surgeon General [11, 14] have confirmed and provided additional evidence for these conclusions.

In the US, the prevalence of cigarette smoking has been substantially different for men and women during the last 50 years. In 1965 the prevalence of smoking was 52.0% and 34.1% for men and women, respectively. In 2017, the corresponding numbers were 15.8% and 12.2%. Also, the prevalence of former smokers peaked in 1985 for men at 30.9%, while this peak was in 1994 at 20% for women [11]. Since the rise and fall in female smoking lagged behind that in men, the rise and fall in smoking-related cancers in women are also expected to emerge later than that in men [15].

A large Norwegian cohort, with follow-up until 2013, suggest that women are more susceptible than men to lung cancer given the same smoking exposure [16]. We have reported possible differences by sex in the smoking-related association with colon and rectal cancer in the Multiethnic Cohort (MEC) study [17]. A difference by sex in the smoking-related association with PC has not been established. The purpose of the present study was to examine whether the detrimental smoking-related association with PC risk is the same for women as for men in the MEC.

## Methods

### Study sample

The MEC consists of more than 215,000 men and women who were aged 45–75 years and living in Los Angeles County, California, and Hawaii at time of cohort recruitment. It comprises mainly five racial/ethnic populations: African American, Japanese American, Latino, Native Hawaiian, and White. Briefly, between 1993 and 1996, participants born between 1918 and 1948 enrolled in the study by completing a 26-page mailed questionnaire asking detailed information about demographic factors, dietary habits, other lifestyle factors, prior medical conditions, and family history of common cancers. We identified potential participants through driver’s license files from the state Departments of Motor Vehicles, voter registration lists, and the Health Care Financing Administration (Medicare) data files. The Institutional Review Boards of the University of Hawaii and the University of Southern California approved the study. The cohort has been previously described in detail [18].

Of the over 215,000 MEC participants, those who did not belong to one of the five main racial/ethnic groups (*n* = 12,206) had prior PC reported on the baseline questionnaire (*n* = 31) or from tumor registries (*n* = 27), without follow-up (*n* = 8), who had implausible diet information (*n* = 8,295), or had missing information on smoking status (*n* = 3,032) were excluded, leaving 192,035 participants (54.6% women) and 1,936 PC cases after a mean follow-up of 19.2 (SD 6.6) years in the analytical cohort. The multivariable analysis used a complete case approach which further excluded subjects with missing data on any of the covariates (*n* = 2,378 including 23 cases), leaving 189,657 (54.4% women) participants and 1,913 PC cases for these analyses.

### Exposure information

At baseline, participants reported whether they had ever smoked at least 20 packs of cigarettes in their lifetime, age at smoking initiation (≥ 25, 20–24, < 20 years), the number of years they smoked cigarettes (≤ 20, 21–30, ≥ 31 years), the average number of cigarettes smoked per day during the period when they smoked(≤ 10, 11–20, ≥ 21), and if they quit smoking, the number of years since they quit (≤ 5, 6–10, 11–15, 16–20, ≥ 21 years) and their age at smoking cessation (≥ 60, 50–59, 40–49, < 40 years). We calculated pack-years as number of cigarettes smoked per day, divided by 20 and multiplied by the duration of smoking in years.

The baseline questionnaire also asked about years of education, height, and current weight for calculating body mass index (BMI in kg/m^2^), physical activity (numbers of hours per day spent in moderate and heavy work or recreational activities), and, for women, age at and type of menopause, and ever use of menopausal hormone therapy. Dietary intake during the previous year was assessed at baseline by a self-administered quantitative food frequency questionnaire with over 180 food items, including alcoholic beverages [18]. We calculated mean alcohol intake in g/day based on the alcohol content of different beverages and usual portion sizes. We calculated daily intakes of nutrients from the questionnaire using a food composition table that has been developed and maintained by the University of Hawaii Cancer Center for the MEC.

### Follow-up and endpoints

We identified incident exocrine PC by linkage to the Surveillance, Epidemiology, and End Results Program cancer registries covering Hawaii and California. We classified PC cases according to anatomical subsites using International Classification of Disease-O3 codes: C25.0 for malignant neoplasm of pancreas, C25.1 for malignant neoplasm of body of pancreas, C25.2 for malignant neoplasm of tail of pancreas, C25.3 for malignant neoplasm of pancreatic duct, C25.7 for malignant neoplasm of other parts of pancreas, C25.8 for malignant neoplasm of overlapping sites of pancreas, and C25.9 for malignant neoplasm of pancreas, unspecified. We did not include C25.4 (*n* = 3) for malignant neoplasm of endocrine pancreas as cases but censored these participants at the date of diagnosis. We classified the tumors according to their stage (localized, regional, distant, unknown). We identified deaths by linkage to death certificate files in Hawaii and California and to the National Death Index. Case and death ascertainment were complete through December 31, 2017. We calculated person-years from cohort entry to the date of PC diagnosis, death, or the end of follow-up (December 31, 2017), whichever occurred first.

### Statistical analysis

We calculated sex-specific PC incidence rates per 100,000 person-years, truncated to ages 45–84 years, and age adjusted to the 2000 US standard population [19]. Subsequently, we calculated corresponding incidence rates for the three (never, former, current) smoking status categories for non-drinkers and drinkers of alcohol and for the five race/ethnicity groups. For each sex, we used Cox proportional hazards regression to model time to PC, with age as the underlying time scale. We computed hazard ratios (HRs) with 95% confidence intervals (CIs) for the associations of PC with different measures of smoking exposure [smoking status at cohort entry (never, former, current); and among former and current smokers, age at smoking initiation (≥ 25, 20–24, < 20 years), smoking duration (≤ 20, 21–30, ≥ 31 years), number of cigarettes smoked per day (≤ 10, 11–20, ≥ 21), and number of pack-years (≤ 10, 11–20, ≥ 21)], using never smokers as the reference group. For former smokers, we computed HRs with 95% CIs for the association between years since quit smoking (≤ 5, 6–10, 11–15, 16–20, ≥ 21 years) and age at smoking cessation (≥ 60, 50–59, 40–49, < 40 years) and PC, using current smokers as the reference group. We computed multivariable-adjusted risk of PC for former and never smokers, with current smokers as reference group, according to years since quit smoking overall and by race/ethnicity, sex, alcohol drinking, and BMI. We used three models to describe the associations between former vs. never, current vs. never, and former vs. current.

We performed lag-time analyses, which excluded incident PC cases within the first 2 and 5 years of follow-up, for current and former smokers according to the different smoking measurers. We did the same lag-time analyses according to tumor status (non-distant and distant tumor status).

In the multivariate analyses, we included as covariates: race/ethnicity (African American, Japanese American, Latino, Native Hawaiian, and White, adjusted as a strata variable), age at cohort entry (continuous), family history of PC (yes, no), history of diabetes (yes, no), and BMI (< 25, 25– < 30, ≥ 30 kg/m^2^). In addition, adjustment was also done for the following dietary intakes per day: alcohol consumption (g) and red/processed meat (g/1,000 kcal). We modeled the dietary intake variables in the multivariate Cox models as continuous variables. We present the results from the complete case analyses throughout the paper. The proportional hazards assumption was tested using Schoenfeld residuals and was found to hold [20, 21]. We conducted tests for linear trends by including an ordinal exposure variable with equally spaced ordinal scores (i.e., 1, 2, 3) in models and never smokers as the first category. We assessed heterogeneity in the PC and smoking association by subgroup based on the Wald statistics for the cross-product terms of subgroup indicator variables and smoking trend variables.

We examined changes in smoking status between enrollment and 10-year follow-up among participants who answered both surveys. We performed the analyses using SAS version 9.4 (SAS Institute Inc., Cary, NC).

## Results

During a mean follow-up of 19.2 years, we identified 1,936 incident PC cases. For men and women, the annual age-adjusted incidence rate of PC was 30.6 and 26.2 per 100,000 person-years (truncated to ages 45–84), respectively*.* For both men and women, Native Hawaiians had the highest (46.4 and 43.1) and Latinos had the lowest (25.6 and 17.0) rates. At enrollment, more than half (52.0%) of the men were former smokers, while 18.1% were current and 29.9% never smokers. For women 30.0% were former, 14.4% current, and more than half (55.7%) never smokers. Among former smokers, 66.7% of men and 58.7% of women had quit smoking > 10 years ago. Among former smokers in the five race/ethnicity groups, most Japanese Americans (68.2%), fewest African Americans (55.4%), and more than 60% among the Latinos, Native Hawaiians, and Whites had quit smoking > 10 years ago.

Table 1 shows that the average age at diagnosis for current smokers was 72.0 years for both men and women. For both sexes, age at diagnosis was lower for current compared with former and never smokers. The proportions of distant-stage tumors among never and current smokers were larger for men than women. The age-adjusted incidence rate of PC among smokers ranged from 14.2 among female Latina former smokers to 85.6 among Native Hawaiian male current smokers, per 100,000 person-years. Among both current and former smokers, compared with their female counterparts, males had initiated smoking at an earlier age, smoked for more years, smoked more cigarettes per day and, consequently, had smoked more pack-years (Table 1).

**Table 1:** Selected characteristics for men and women at baseline in 1993–1996, according to smoking status in the multiethnic cohort study, followed to 2017

Characteristics	Men (*n* = 87,129)			Women (*n* = 104,906)			Total
	Never	Former	Current	Never	Former	Current	
No. of participants	26,038	45,321	15,770	58,501	31,414	14,991	192,035
Age at cohort entry (years, SD)	59.5 (9.1)	61.4 (8.6)	58.3 (8.6)	60.3 (8.9)	59.8 (8.7)	57.5 (8.5)	60.0 (8.8)
Follow-up years (SD)	19.7 (6.2)	18.1 (7.1)	16.9 (7.5)	20.5 (5.7)	19.4 (6.5)	18.4 (7.0)	19.2 (6.6)
No. of pancreatic cancer cases	275	437	186	595	283	160	1936
Age at diagnosis (years, SD)	76.4 (8.7)	76.2 (8.8)	72.0 (8.9)	78.0 (8.2)	75.3 (8.5)	72.0 (8.6)	75.9 (8.8)
Stage of tumor (%)							
Localized	9.5	10.3	7.0	8.6	6.7	9.4	8.7
Regional	28.4	27.0	26.9	31.4	28.6	31.3	29.1
Distant	47.3	49.2	52.2	42.9	50.5	45.0	47.1
Unknown	14.9	13.5	14.0	17.1	14.1	14.4	15.0
Incidence/100,000^a^							
All	27.1	26.6	48.0	22.6	26.5	38.7	28.2
Non-drinkers	31.5	25.2	55.9	24.2	28.1	41.2	28.9
Drinkers	23.9	27.8	45.0	19.6	24.6	36.7	27.6
African Americans	23.9	23.8	65.8	22.9	38.2	53.1	35.0
Native Hawaiians	41.9	33.4	85.6	44.2	40.7	51.8	44.6
Japanese Americans	31.2	28.9	48.8	23.7	32.5	46.7	30.0
Latinos	21.2	25.1	33.9	17.5	14.2	19.3	20.9
Whites	25.6	23.6	36.3	20.1	17.5	30.2	23.1
Age at smoking initiation (years, SD)	N/A	27.0 (9.8)	26.5 (10.3)	N/A	29.7 (10.6)	28.4 (10.9)	27.9 (10.4)
Smoking duration (years, SD)	N/A	20.4 (12.0)	31.9 (9.8)	N/A	17.4 (11.7)	29.1 (10.3)	22.5 (12.6)
No. of cigarettes smoked per day (SD)	N/A	16.1 (8.5)	16.3 (8.2)	N/A	12.2 (7.7)	13.9 (7.5)	14.7 (8.3)
Pack-years of smoking (SD)	N/A	18.5 (16.1)	27.0 (16.7)	N/A	12.5 (13.3)	21.3 (14.8)	18.4 (15.9)
Age at smoking cessation (years, SD)	N/A	47.3 (10.1)	N/A	N/A	47.1 (10.7)	N/A	47.2 (10.3)
Family history of pancreatic cancer (%)	1.4	1.5	0.9	2.0	2.1	1.5	1.7
History of diabetes (%)	10.7	14.4	11.0	10.9	12.5	9.7	11.9
Body mass index (%)							
< 25 kg/m^2^	37.2	33.4	41.9	48.1	41.6	47.9	41.5
25‒29.9 kg/m^2^	46.7	48.4	42.6	31.5	32.7	31.6	38.7
≥ 30 kg/m^2^	16.1	18.3	15.5	20.4	25.7	20.6	19.8
Alcohol consumption (g/day, SD)	9.6 (23.8)	14.6 (31.3)	23.2 (45.3)	2.3 (9.4)	5.6 (15.6)	9.4 (25.6)	9.0 (25.2)
Red/processed meat intake (g/1000 kcal, SD)	28.1 (16.6)	29.3 (16.7)	35.3 (17.6)	23.9 (15.3)	24.5 (16.0)	29.9 (17.0)	27.3 (16.6)

*SD* standard deviation

^a^Rates, truncated to ages 45–84, were adjusted to the 2000 US standard population

Table 2 shows that the age- and multivariable-adjusted HR estimates for PC risks are quite similar. Compared with never smokers, current smokers had a 51% (HR 1.51, 95% CI 1.32, 1.73) overall higher risk of PC after multivariable adjustment, while former smokers (HR 0.94, 95% CI 0.84, 1.05) did not. All of the 22 HR estimates for former smokers with never smokers as a reference group were non-significant and only one estimate was above 1.0. Former smokers who had quitted less than five years showed a 20% (HR 0.80, 95% CI 0.66, 0.97) and those who had quitted before the age of 60 years a 34% (HR 0.66, 95% CI 0.55, 0.80) decrease in PC risk, compared with current smokers (Table 2).

**Table 2:** Age- and multivariable-adjusted HR estimates for pancreatic cancer in former and current smokers in the MEC, 1993–2017

Smoking exposures	Former smokers (*n* = 76,735)				Current smokers (*n* = 30,761)			
	Cases	HR (95% CI)^a^	Cases^b^	HR (95% CI)^c^	Cases	HR (95% CI)^a^	Cases^b^	HR (95% CI)^c^
Common reference group								
Never smokers	870	1.00 (ref)	856	1.00 (ref)	870	1.00 (ref)	856	1.00 (ref)
Smokers	720	0.96 (0.86–1.07)	714	0.94 (0.84–1.05)	346	1.47 (1.29–1.68)	343	1.51 (1.32–1.73)
Age at smoking initiation								
≥ 25 years	425	0.95 (0.84–1.08)	421	0.94 (0.83–1.06)	190	1.41 (1.20–1.66)	188	1.46 (1.24–1.72)
20–24 years	136	1.00 (0.83–1.20)	136	0.97 (0.81–1.18)	89	1.61 (1.28–2.01)	88	1.63 (1.30–2.05)
< 20 years	129	0.94 (0.78–1.14)	128	0.90 (0.74–1.10)	63	1.54 (1.17–2.01)	63	1.57 (1.19–2.06)
*p*_trend_^d^		0.54		0.29		< 0.001		< 0.001
Smoking duration								
≤ 20 years	375	0.93 (0.82–1.05)	371	0.91 (0.81–1.04)	53	1.43 (1.08–1.89)	53	1.48 (1.12–1.96)
21–30 years	153	0.93 (0.78–1.11)	152	0.91 (0.76–1.08)	83	1.54 (1.22–1.94)	82	1.57 (1.24–1.99)
≥ 31 years	168	1.09 (0.92–1.30)	168	1.06 (0.89–1.27)	206	1.47 (1.25–1.72)	204	1.51 (1.28–1.78)
*p*_trend_^d^		0.76		0.96		< 0.001		< 0.001
Number of cigarettes								
≤ 10/day	302	0.94 (0.82–1.07)	299	0.93 (0.82–1.07)	122	1.29 (1.06–1.57)	121	1.34 (1.10–1.63)
11–20/day	249	1.02 (0.88–1.18)	247	0.99 (0.85–1.15)	133	1.50 (1.25–1.81)	131	1.54 (1.27–1.87)
≥ 21/day	143	0.89 (0.74–1.07)	142	0.84 (0.70–1.02)	85	1.75 (1.38–2.20)	85	1.80 (1.42–2.28)
*p*_trend_^d^		0.38		0.15		< 0.001		< 0.001
Pack-years								
≤ 10	281	0.93 (0.81–1.07)	278	0.92 (0.80–1.06)	54	1.30 (0.98–1.72)	54	1.35 (1.02–1.79)
11–20	222	0.97 (0.83–1.13)	221	0.95 (0.81–1.11)	117	1.39 (1.14–1.69)	116	1.44 (1.18–1.76)
≥ 21	177	0.98 (0.82–1.16)	176	0.93 (0.78–1.11)	166	1.60 (1.35–1.91)	164	1.64 (1.38–1.97)
*p*_trend_^d^		0.64		0.33		< 0.001		< 0.001
Years since quit								
Current smokers	346	1.00 (ref)	343	1.00 (ref)				
≤ 5 years	149	0.81 (0.67–0.99)	149	0.80 (0.66–0.97)				
6–10 years	118	0.70 (0.57–0.86)	117	0.68 (0.55–0.84)				
11–15 years	105	0.63 (0.51–0.78)	103	0.61 (0.49–0.76)				
16–20 years	95	0.61 (0.48–0.76)	94	0.60 (0.47–0.75)				
≥ 21 years	247	0.60 (0.51–0.71)	245	0.60 (0.50–0.71)				
*p*_trend_		< 0.001		< 0.001				
Age at smoking cessation								
Current smokers	346	1.00 (ref)	343	1.00 (ref)				
≥ 60 years	124	0.92 (0.74–1.15)	123	0.91 (0.72–1.14)				
50–59 years	230	0.68 (0.57–0.81)	228	0.66 (0.55–0.80)				
40–49 years	242	0.62 (0.52–0.73)	240	0.61 (0.51–0.72)				
< 40 years	118	0.60 (0.48–0.74)	117	0.59 (0.48–0.74)				
*p*_trend_		< 0.001		< 0.001				

*CI* confidence interval, *HR* hazard ratio

^a^Adjusted for age at cohort entry, sex, and race/ethnicity

^b^Excluding participants with missing information on covariates

^c^Further adjusted for family history of pancreatic cancer, history of diabetes, body mass index, alcohol consumption, and red/processed meat intake

^d^Based on equally spaced ordinal scores with never smokers as the first category

Supplemental Table 1 shows lag-time analyses, which excluded incident cases within the first two and five years of follow-up, for current and former smokers according to the different smoking measurers. For both former and current smokers, the results are similar to the results in Table 2, overall and for the displayed smoking measures (Supplemental Table 1).

Current smokers had an overall 39% (HR 1.39, 95% CI 1.12, 1.72) higher risk of non-distant and a 59% (HR 1.59, 95% CI 1.31, 1.72) higher risk of distant PC compared with never smokers. The results did not change materially from the overall results when we did the same lag-time (2 and 5 years) analyses according to non-distant and distant tumor status (data not shown).

Table 3 shows that former smokers had for both men, HR 0.90 (95% CI 0.77, 1.05) and women, HR 0.99 (95% CI 0.86, 1.15) an overall similar non-significant reduction in risk of PC, after multivariable adjustments, compared with never smokers. Current smokers had an overall similar higher risk of PC for both men, HR 1.48 (95% CI 1.22, 1.79) and women, HR 1.49 (95% CI 1.24, 1.79) compared with never smokers.

**Table 3:** Smoking and pancreatic cancer risk in current and former smokers by sex in the MEC, 1993–2017

Smoking exposures	Former smokers					Current smokers				
	Men		Women		*p*_heterogeneity_	Men		Women		*p*_heterogeneity_
	Cases	HR (95% CI)^a^	Cases	HR (95% CI)^a^		Cases	HR (95% CI)^a^	Cases	HR (95% CI)^a^	
Common reference group										
Never smokers	274	1.00 (ref)	582	1.00 (ref)		274	1.00 (ref)	582	1.00 (ref)	
Smokers	435	0.90 (0.77–1.05)	279	0.99 (0.86–1.15)	0.36	184	1.48 (1.22–1.79)	159	1.49 (1.24–1.79)	0.79
Age at smoking initiation										
≥ 25 years	244	0.91 (0.76–1.08)	177	0.95 (0.80–1.13)		96	1.50 (1.18–1.91)	92	1.42 (1.13–1.78)	
20–24 years	87	0.90 (0.70–1.14)	49	1.08 (0.80–1.46)		49	1.62 (1.18–2.22)	39	1.66 (1.18–2.32)	
< 20 years	86	0.81 (0.64–1.04)	42	1.07 (0.78–1.48)		36	1.49 (1.03–2.15)	27	1.71 (1.14–2.57)	
*p*_trend_^b^		0.087		0.73	0.17		< 0.001		< 0.001	0.64
Smoking duration										
≤ 20 years	216	0.89 (0.74–1.06)	155	0.93 (0.77–1.11)		25	1.60 (1.06–2.43)	28	1.39 (0.94–2.04)	
21–30 years	98	0.85 (0.67–1.07)	54	0.96 (0.73–1.28)		35	1.44 (1.00–2.08)	47	1.69 (1.24–2.30)	
≥ 31 years	108	0.94 (0.75–1.18)	60	1.25 (0.95–1.64)		121	1.54 (1.23–1.92)	83	1.47 (1.16–1.86)	
*p*_trend_^b^		0.36		0.41	0.25		< 0.001		< 0.001	0.99
Number of cigarettes										
≤ 10/day	141	0.88 (0.72–1.08)	158	0.97 (0.81–1.16)		58	1.46 (1.09–1.95)	63	1.25 (0.96–1.63)	
11–20/day	167	0.95 (0.78–1.15)	80	1.04 (0.82–1.31)		64	1.38 (1.04–1.83)	67	1.73 (1.33–2.26)	
≥ 21/day	108	0.79 (0.63–0.99)	34	0.92 (0.65–1.32)		58	1.82 (1.35–2.46)	27	1.71 (1.15–2.55)	
*p*_trend_^b^		0.075		0.84	0.34		< 0.001		< 0.001	0.77
Pack-years										
≤ 10	139	0.87 (0.71–1.06)	139	0.97 (0.80–1.17)		28	1.67 (1.12–2.49)	26	1.12 (0.75–1.67)	
11–20	151	0.95 (0.78–1.16)	70	0.90 (0.70–1.15)		50	1.33 (0.98–1.81)	66	1.54 (1.18–2.00)	
≥ 21	121	0.82 (0.66–1.02)	55	1.18 (0.89–1.57)		99	1.59 (1.25–2.02)	65	1.71 (1.31–2.24)	
*p*_trend_^b^		0.11		0.79	0.23		< 0.001		< 0.001	0.64
Years since quit										
Current smokers	184	1.00 (ref)	159	1.00 (ref)						
≤ 5 years	77	0.74 (0.57–0.97)	72	0.87 (0.66–1.15)						
6–10 years	67	0.66 (0.50–0.88)	50	0.70 (0.50–0.96)						
11–15 years	64	0.60 (0.45–0.80)	39	0.61 (0.43–0.87)						
16–20 years	58	0.56 (0.42–0.76)	36	0.64 (0.44–0.92)						
≥ 21 years	164	0.57 (0.46–0.71)	81	0.63 (0.48–0.83)						
*p*_trend_		< 0.001		< 0.001	0.78					
Age at smoking cessation										
Current smokers	184	1.00 (ref)	159	1.00 (ref)						
≥ 60 years	71	0.86 (0.64–1.16)	52	0.95 (0.67–1.34)						
50–59 years	136	0.61 (0.48–0.78)	92	0.74 (0.56–0.97)						
40–49 years	151	0.58 (0.46–0.72)	89	0.64 (0.49–0.84)						
< 40 years	72	0.59 (0.44–0.79)	45	0.59 (0.42–0.84)						
*p*_trend_		< 0.001		< 0.001	0.67					

*CI* confidence interval, *HR* hazard ratio

^a^Adjusted for age at cohort entry, race/ethnicity, family history of pancreatic cancer, history of diabetes, body mass index, alcohol consumption, and red/processed meat intake

^b^Based on equally spaced ordinal scores with never smokers as the first category

For men former smokers compared with never smokers, the four (age at smoking initiation, smoking duration, number of cigarettes per day, number of pack-years) measures of smoking exposure displayed showed no direct associations with risk of PC with all HR estimates below 1.0 and no dose–response (all *p*_trend_’s > 0.07). Women former smokers showed non-statistically significant associations for those with the longest (≥ 31 years) smoking duration (HR 1.25, 95% CI 0.95, 1.64) and the highest (≥ 21) number of pack-years (HR 1.18, 95% CI 0.89, 1.57). Former smokers showed within 5 years after quitting, a 26% (HR 0.74, 95% CI 0.57, 0.97) decrease in risk of PC for men and within 10 years after quitting, a 30% (HR 0.70, 95% CI 0.50, 0.96) decrease in risk of PC for women, compared with their sex-specific current smokers. Former smokers who quitted before the age of 60 showed for men a 39% (HR 0.61, 95% CI 0.48, 0.78) and for women a 26% (HR 0.74, 95% CI 0.56, 0.97) decrease in risk of PC, compared with their sex-specific reference group. For former smokers, both men and women showed a strong inverse dose–response association with PC risk and years since quit smoking and age at smoking cessation (all *p*_trend_’s < 0.001).

Current smokers who had initiated smoking before 20 years of age, had for men a 49% (HR 1.49, 95% CI 1.03, 2.15), and for women a 71% (HR 1.71, 95% CI 1.14, 2.57) higher risk of PC, compared with their sex-specific never smokers. Current smokers showed, for both men and women, significantly higher risk of PC for most of the displayed measures of smoking exposure, compared with their sex-specific reference groups. Current smokers had, for both sexes, a similar strong direct dose–response association for the four (age at smoking initiation, smoking duration, number of cigarettes per day, number of pack-years) measures of smoking exposure (all *p*_trend_’s < 0.001) (Table 3).

Supplemental Table 2 shows that current smokers had for African Americans a 48% (HR 1.48, 95% CI 1.11, 1.97) and for Japanese Americans an 82% (HR 1.82, 95% CI 1.44, 2.32) higher risk of PC, compared with never smokers. The Native Hawaiian, Latino, and White groups had similar non-statistically significant risks of PC among current compared with never smokers (Supplemental Table 2).

**Fig. 1:**
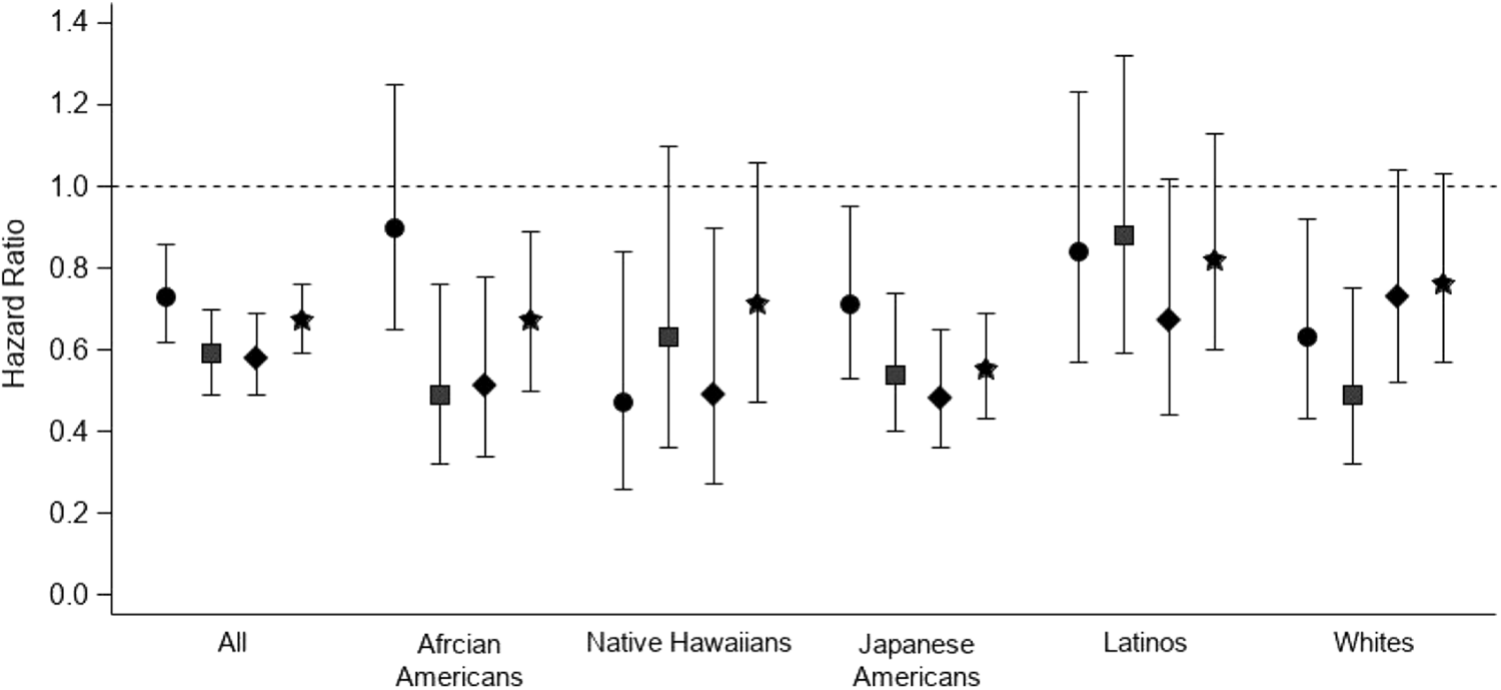
Multivariable-adjusted risk of pancreatic cancer for former and never smokers, with current smokers as reference group, according to years since quit smoking overall and by race/ethnicity in the MEC, 1993–2017. Results were adjusted for age at cohort entry, sex, race/ethnicity, family history of pancreatic cancer, history of diabetes, body mass index, alcohol consumption, and red/processed meat intake appropriately. Circles, ≤ 10 years; squares, 11–20 years; diamonds, ≥ 21 years since quit smoking; stars, never smokers. Bars, 95% confidence intervals

Figure 1 shows that compared with current smokers, former smokers had within the first decade after quitting a significant reduction in overall PC risk. This risk was approaching that of never smokers, with overlapping CI’s. After stratification the associations were less consistent, but still show a similar pattern for the association between time since quit smoking and PC risk in the five race/ethnicity groups (Fig. 1).

**Fig. 2:**
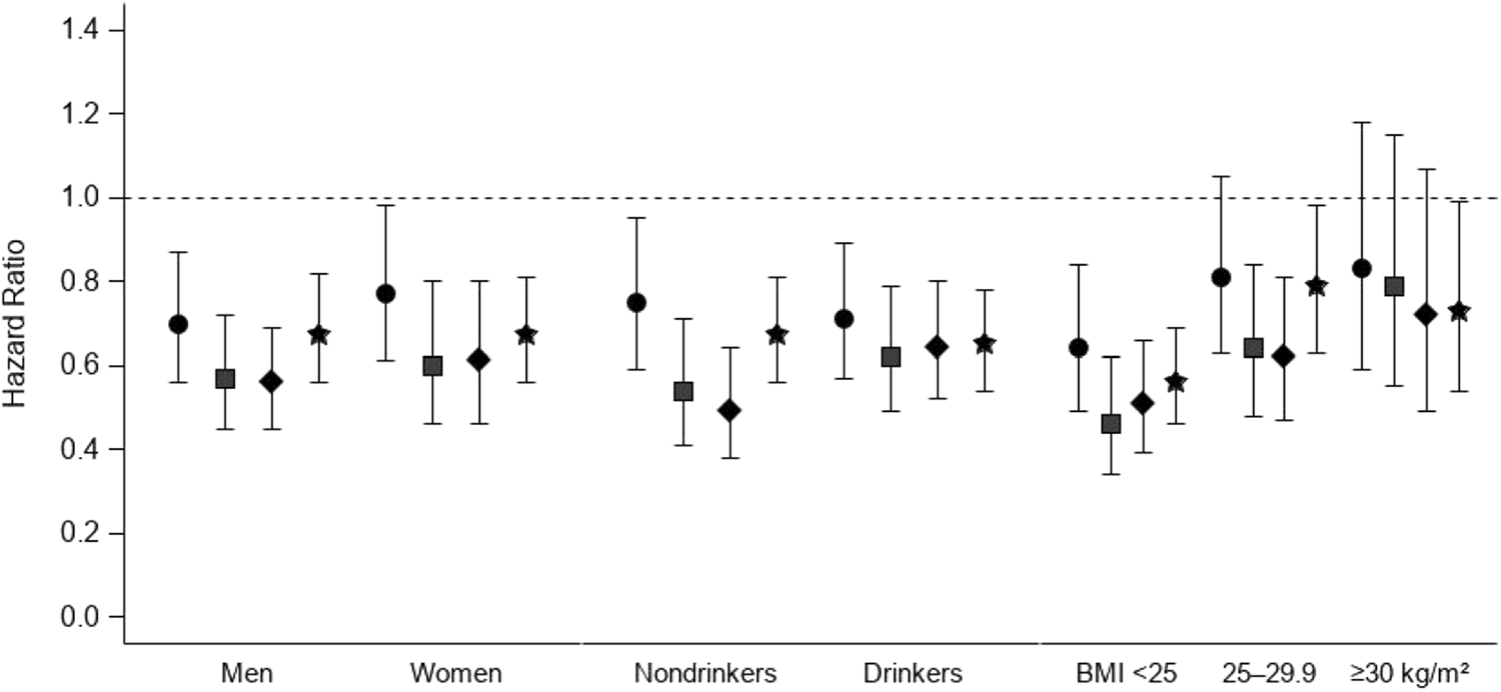
Multivariable-adjusted risk of pancreatic cancer for former and never smokers, with current smokers as reference group, according to smoking status and years since quit smoking in subgroups by sex, alcohol drinking, and body mass index (BMI), in the MEC, 1993–2017. Results were adjusted for age at cohort entry, sex, race/ethnicity, family history of pancreatic cancer, history of diabetes, BMI, alcohol consumption, and red/processed meat intake appropriately. Circles, ≤ 10 years; squares, 11–20 years; diamonds, ≥ 21 years since quit smoking; stars, never smokers. Bars, 95% confidence intervals

Figure 2 shows that compared with current smokers, former smokers had within the first decade after quitting a significant reduction in PC risk, for men, women, non-drinkers, drinkers of alcohol, and those with a BMI less than 25. For the listed subgroups, this PC risk was approaching that of never smokers, with overlapping CI’s (Fig. 2).

Altogether 89,006 (46.3%) participated in the 10-year follow-up survey, of which 2.6% men and 2.8% had missing on smoking status. All of the never smokers and among former smokers 98.7% men and 98.6% women reported no change in smoking status. Among the current smokers, 3,357 (59%) men and 3,387 (57.5%) women reported quitting smoking during this 10-year period (data not shown).

## Discussion

This study is, to our knowledge, the first large prospective cohort that examines in detail the association between smoking and PC risk by sex. Compared with never smokers, former smokers did not have an increase in risk of PC, while current smokers had a consistent, strong, dose–response-elevated PC risk for the four measures of smoking exposure examined (age at smoking initiation, smoking duration, number of cigarettes per day, and pack-years). The detrimental smoking-related association with PC risk was the same for women as for men, for both former and current smokers. Even so, compared with continued smoking, men had a significantly decrease in PC risk within 5 years of quitting, while women did not. There was a significant inverse dose–response association with number of years of quitting and age at smoking cessation for both sexes. Compared with continued smoking, within 10 years of quitting, the risks of PC were like that of never smokers for former smokers overall, for both sexes, non-drinkers and drinkers of alcohol, and for those with a normal BMI. Furthermore, for all five racial/ethnic groups, the smoking-related risk of PC for current smokers was increased similarly compared with never smokers, as was the decrease according to years since quitting compared with continued smoking.

Our results are consistent with those of several recent cohort studies showing that cigarette smoking is associated with increased risk of PC among current smokers [22–29], while former smokers do not have an increase in risk [22–24, 26–29] and that the risk is reduced to levels of never smokers within 5 to 10 years [22, 23, 26, 27].

Only four of these cohorts [22–24, 27], which all had less than 225 PC cases, displayed results by sex. Both the Norwegian [22] and one of the Swedish studies [23] found that the associations with pack-years of smoking for both former and current smokers were stronger for women than men. The other study from Sweden found for women, but not for men, statistically significant doubling in risks of PC for occasional smoking and for exposure to environmental smoke at work for > 20 years [27].

Three recent studies performing large meta-analysis did not report sex-specific analyses for the association between smoking and PC [30–32], while three others did [33–35]. In contrast to our findings, two [30, 31] of the former, which included both case–control and cohort studies, showed significantly increased risks for former smokers, while the study from the Pancreatic Cancer Cohort Consortium did not. In this study, Lynch et al. reported that they did not find any heterogeneity by sex [32]. The study by Zou et al. also included both case–control and cohort studies. They found a non-linear dose–response associations for all metrics of cigarette smoking in women, while linear relationships were observed for smoking duration and cumulative number of cigarettes smoked in men, except for smoking intensity. The authors concluded that this non-linear dose–response relationship between smoking and PC risk might differ by sex [33].

The Australian study [34] including seven cohorts and the Japanese study including ten population-based cohort studies [35] both found some indications of a possible sex difference for the smoking-related association with PC. The Australian study comprising 604 incident PC cases found that the risk associated with current smoking was greater for men than for women. Both sexes had a similar significant increase in risk for former smokers who had stopped < 15 years ago, which disappeared after ≥ 15 years [34]. The Japanese study comprising 1779 incident PC cases found that the risk associated with current smoking was similar for men and women. However, it was only among women they observed a significant elevation in risk for former smokers. For men they found a significant decrease in risk after 5 years of smoking cessation, while the women had an increased risk of PC also ≥ 10 after cessation [35].

The MEC study includes the birth cohorts of men and women that have had the highest smoking prevalence across time in the USA. [11, 14]. Already in 1983, Weiss and Benarde suggested that we should expect an increasing trend of PC for women and a decreasing trend in PC for men, based on the difference in smoking histories [36]. Our results support this expectation, as the detrimental smoking-related association with PC risk is the same for women as for men, for both former and current smokers.

Major strengths of our study include the large sample size (close to 2000 PC cases), its focus on the smoking-related risk of PC by sex, the multiethnic population, and the use of several measures of smoking exposure among current smokers and years since and age at quitting among former smokers. We have a high proportion of current and former smokers in both sexes. We obtained the smoking histories at enrollment; thus, our results are unlikely to be subject to recall bias. Our minimum enrollment age was 45 years and, in the USA, hardly anyone starts to smoke after age 50 years [11]. We were able to examine, among the close to 50% that participated in our 10-year follow-up survey, that none of the never and almost none of the former smokers had changed smoking status.

We were able to classify 85% of the tumors according to their stage at diagnosis. Also, the results were materially the same in the lag-time analyses we performed. Furthermore, we have demonstrated the internal validity of the smoking exposure variables [17, 29, 37–40] and associations with family history of PC, diabetes, and lifestyle factors (smoking, BMI, red meat intake and alcohol consumption) and the PC outcome [29]. We have detailed information on, and were able to control for, the established risk factors for PC, many of which vary according to smoking status.

The main limitation of this study is that despite the large number of incident PC cases, the numbers of cases were small for certain subset analyses. Nevertheless, our study shows consistent associations for several subgroup analyses. The lack of a statistical significance for the association with current smoking among Native Hawaiians, Latinos, and Whites is likely a function of power rather than strength of the association.

Another limitation is that the current smokers that had quitted smoking are incorrectly in the current smoker category and will attenuate the association. Also, we lack information about childhood and adult passive smoking, which both have been associated with increased risk of PC [26, 27]. More women were never smokers and participants exposed to passive smoking were included in the sex-specific reference groups. Thus, our PC risk estimates could have been attenuated more for women than for men. Another limitation is that our questionnaire did not use open-ended questions for cigarettes per day, smoking duration, and duration of quitting. The previously mentioned Norwegian study of sex differences in the smoking and lung cancer association had more than 6,500 cases. However, it was only when the smoking exposure was analyzed as a continuous variable that female current smokers had a significantly higher risk of lung cancer than male current smokers for increments of pack-years, cigarettes per day, and smoking duration [16]. In the MEC cohort, for those checking the same exposure category, most likely, more women will be in the lower and more men in the higher end of the selected category range. Current smokers will consequently have underestimated PC risk for women and overestimated PC risk for men in the same smoking exposure category.

More men than women were smokers in the present study. Consequently, more men than women have died from different causes of smoking-related deaths before they were diagnosed with PC. Also, smoking as well other risk factors were self-reported. Since the questionnaire was filled in at enrollment and before case identification, we expect any misclassification to be non-differential and bias our results toward the null. We lack information about chronic pancreatitis, a rare, but established risk factor for pancreas cancer [41]. Most of the abovelisted limitations may attenuate or conceal a possible sex difference of the smoking-related association with PC risk.

Smoking cigarettes causes exposure to a mixture of more than 8,000 compounds, including more than 70 known carcinogens. Cigarette smoking causes at least 20 different types or subtypes of cancer risk [42]. Smoking contributes to carcinogenesis through multiple biological mechanisms, which collectively can act at the early and late stages of carcinogenesis. Regardless of the specific mechanisms, smoking cessation ends further increments to cumulative exposure to tobacco smoke and, therefore, is expected to reduce the risk of cancer caused by smoking. For PC the evidence available for the most recent US Surgeon General report indicated that former smokers would approach that of never smokers approximately 20 years after smoking cessation [11].

In the MEC study, although women former and current smokers smoke on average less than their men counterparts, the smoking-related increase in PC risk and the benefits of cessation seem to be of similar magnitude for women as for men.

## Supplementary Information

Below is the link to the electronic supplementary material.

Supplementary file1 (DOCX 30 KB)

## Data Availability

For information on how to gain access to data from the multiethnic cohort, please see: https://www.uhcancercenter.org/for-researchers/mec-data-sharing.

## Abbreviations

BMI: Body mass index
CI: Confidence interval
HR: Hazard ratio
MEC: Multiethnic Cohort
PC: Pancreatic cancer
