# Supplementary material for: Smoking and pancreatic cancer: a sex-specific analysis in the Multiethnic Cohort study
Source: Cancer Causes Control. 2022 Oct 17;34(1):89–100. doi: 10.1007/s10552-022-01637-z (PMC9816198; doi:10.1007/s10552-022-01637-z)
Supplement: Supplementary file 1 — Supplementary file1 (DOCX 30 KB) [file 10552_2022_1637_MOESM1_ESM.docx]

**Supplemental Table 1.** Lag-time analysis of Smoking and pancreatic cancer in former and current smokers in the MEC, 1993-2017

|  | 2-year lagged | | | | 5-year lagged | | | |
| --- | --- | --- | --- | --- | --- | --- | --- | --- |
|  | Former smokers | | Current smokers | | Former smokers | | Current smokers | |
|  | Cases | HR (95% CI)^a^ | Cases | HR (95% CI)^a^ | Cases | HR (95% CI)^a^ | Cases | HR (95% CI)^a^ |
| Common reference group |  |  |  |  |  |  |  |  |
| Never smokers | 825 | 1.00 (ref) | 825 | 1.00 (ref) | 766 | 1.00 (ref) | 766 | 1.00 (ref) |
| Smokers | 667 | 0.93 (0.83-1.03) | 323 | 1.51 (1.32-1.74) | 593 | 0.89 (0.80-1.00) | 287 | 1.46 (1.26-1.69) |
| Age at smoking initiation |  |  |  |  |  |  |  |  |
| ≥25 years | 392 | 0.92 (0.82-1.05) | 176 | 1.47 (1.24-1.74) | 346 | 0.89 (0.78-1.02) | 158 | 1.45 (1.21-1.73) |
| 20-24 years | 128 | 0.97 (0.80-1.17) | 84 | 1.63 (1.29-2.07) | 114 | 0.93 (0.76-1.14) | 75 | 1.56 (1.22-1.99) |
| <20 years | 122 | 0.91 (0.75-1.11) | 60 | 1.56 (1.18-2.07) | 110 | 0.88 (0.71-1.08) | 51 | 1.41 (1.04-1.91) |
| *P*_trend_^b^ |  | 0.28 |  | <0.001 |  | 0.13 |  | <0.001 |
| Smoking duration |  |  |  |  |  |  |  |  |
| ≤20 years | 355 | 0.91 (0.80-1.04) | 52 | 1.54 (1.15-2.04) | 326 | 0.90 (0.79-1.03) | 49 | 1.56 (1.16-2.09) |
| 21-30 years | 136 | 0.86 (0.71-1.03) | 76 | 1.53 (1.20-1.96) | 118 | 0.81 (0.66-0.98) | 72 | 1.56 (1.22-2.01) |
| ≥31 years | 155 | 1.06 (0.88-1.27) | 192 | 1.51 (1.28-1.79) | 130 | 0.99 (0.81-1.20) | 163 | 1.41 (1.18-1.68) |
| *P*_trend_^b^ |  | 0.70 |  | <0.001 |  | 0.22 |  | <0.001 |
| Number of cigarettes |  |  |  |  |  |  |  |  |
| ≤10/day | 281 | 0.92 (0.80-1.05) | 115 | 1.35 (1.11-1.66) | 255 | 0.90 (0.78-1.04) | 106 | 1.35 (1.10-1.67) |
| 11-20/day | 232 | 0.99 (0.85-1.15) | 122 | 1.53 (1.26-1.87) | 204 | 0.94 (0.80-1.11) | 106 | 1.45 (1.17-1.79) |
| ≥21/day | 129 | 0.82 (0.67-0.99) | 80 | 1.78 (1.40-2.28) | 109 | 0.75 (0.61-0.93) | 69 | 1.69 (1.30-2.19) |
| *P*_trend_^b^ |  | 0.095 |  | <0.001 |  | 0.016 |  | <0.001 |
| Pack-years |  |  |  |  |  |  |  |  |
| ≤10 | 265 | 0.92 (0.79-1.05) | 53 | 1.40 (1.06-1.86) | 239 | 0.89 (0.76-1.03) | 49 | 1.39 (1.04-1.87) |
| 11-20 | 204 | 0.93 (0.79-1.09) | 108 | 1.42 (1.16-1.75) | 186 | 0.91 (0.77-1.08) | 101 | 1.44 (1.16-1.79) |
| ≥21 | 161 | 0.92 (0.77-1.10) | 154 | 1.64 (1.37-1.97) | 133 | 0.84 (0.69-1.02) | 129 | 1.51 (1.24-1.84) |
| *P*_trend_^b^ |  | 0.21 |  | <0.001 |  | 0.047 |  | <0.001 |
| Years since quit |  |  |  |  |  |  |  |  |
| Current smokers | 323 | 1.00 (ref) |  |  | 287 | 1.00 (ref) |  |  |
| ≤5 years | 141 | 0.80 (0.66-0.98) |  |  | 121 | 0.76 (0.62-0.95) |  |  |
| 6-10 years | 110 | 0.68 (0.54-0.84) |  |  | 95 | 0.65 (0.51-0.82) |  |  |
| 11-15 years | 97 | 0.61 (0.48-0.76) |  |  | 89 | 0.62 (0.48-0.78) |  |  |
| 16-20 years | 85 | 0.57 (0.45-0.73) |  |  | 73 | 0.54 (0.42-0.70) |  |  |
| ≥21 years | 230 | 0.59 (0.50-0.71) |  |  | 211 | 0.61 (0.50-0.73) |  |  |
| *P*_trend_ |  | <0.001 |  |  |  | <0.001 |  |  |
| Age at smoking cessation |  |  |  |  |  |  |  |  |
| Current smokers | 323 | 1.00 (ref) |  |  | 287 | 1.00 (ref) |  |  |
| ≥60 years | 113 | 0.93 (0.73-1.17) |  |  | 90 | 0.87 (0.67-1.12) |  |  |
| 50-59 years | 210 | 0.66 (0.55-0.80) |  |  | 189 | 0.67 (0.55-0.82) |  |  |
| 40-49 years | 224 | 0.59 (0.50-0.71) |  |  | 201 | 0.58 (0.48-0.70) |  |  |
| <40 years | 116 | 0.60 (0.48-0.75) |  |  | 109 | 0.60 (0.48-0.76) |  |  |
| *P*_trend_ |  | <0.001 |  |  |  | <0.001 |  |  |

Abbreviations: CI, confidence interval; HR, hazard ratio.

^a^Adjusted for age at cohort entry, sex, race/ethnicity, family history of pancreatic cancer, history of diabetes, body mass index, alcohol consumption, and red/processed meat intake.

^b^Based on equally spaced ordinal scores with never smokers as the first category.

**Supplemental Table 2.** Smoking and pancreatic cancer risk by race/ethnicity in the MEC, 1993-2017

| Race/ethnicity | Never smokers | | Former smokers | | Current smokers | |
| --- | --- | --- | --- | --- | --- | --- |
|  | Cases | HR (95% CI)^a^ | Cases | HR (95% CI)^a^ | Cases | HR (95% CI)^a^ |
| African American | 134 | 1.00 (ref) | 130 | 0.99 (0.77-1.26) | 84 | 1.48 (1.11-1.97) |
| Japanese American | 316 | 1.00 (ref) | 246 | 1.04 (0.86-1.25) | 100 | 1.82 (1.44-2.32) |
| Latino | 178 | 1.00 (ref) | 140 | 0.97 (0.76-1.22) | 53 | 1.21 (0.88-1.67) |
| Native Hawaiian | 72 | 1.00 (ref) | 51 | 0.74 (0.51-1.06) | 40 | 1.41 (0.94-2.11) |
| White | 156 | 1.00 (ref) | 147 | 0.82 (0.65-1.03) | 66 | 1.31 (0.97-1.77) |
| *P*_heterogeneity_^b^ |  | 0.32 |  |  |  |  |

Abbreviations: CI, confidence interval; HR, hazard ratio.

^a^Adjusted for age at cohort entry, sex, family history of pancreatic cancer, history of diabetes, body mass index, alcohol consumption, and red/processed meat intake appropriately.

^b^Based on the Wald statistics for the cross-product terms of race/ethnicity indicator variable and smoking trend variable.
